# Antioxidant and Antibacterial Activity of Plant Extracts

**DOI:** 10.3390/antibiotics12071176

**Published:** 2023-07-12

**Authors:** Delia Muntean, Silvana Vulpie

**Affiliations:** Department of Microbiology, “Victor Babes” University of Medicine and Pharmacy, Eftimie Murgu Square, No. 2, 300041 Timisoara, Romania

Medicinal plants have been a very important source of medicinal products for millennia. A significant number of drugs used in the medicine of the 21st century have been developed from vegetal sources. Traditionally, plants have been used for their anti-inflammatory, antioxidant, and antibacterial effects. Identifying, testing, and implementing the use of new molecules in current therapeutic protocols is a very complex, time-consuming, and expensive process. In many situations, attention is directed towards the study of plant extracts as it is very well known that, in some cases, the activity of the phytocomplex is superior to that of pure phytochemicals due to the synergism of molecular structures.

This Special Issue includes two reviews and seven research articles and focuses on the evaluation of chemical compounds from plant extracts and their biological and pharmacological effects, such as antioxidant properties and antibacterial activity. 

Firstly, we started with two interesting reviews in the dermatological field. One of these reviews is an update on the cutaneous benefits of *Origanum vulgare* L. essential oil [1], referring to the potential of this essential oil of being used in the development of modern formulations, such as microemulsions and nanoemulsions, in order to create the possibility for topical application for skin disorders. The other is a review focused on the antioxidative, dermal immunomodulatory, and antimicrobial properties of bark extracts from common European temperate trees [2].

Two more papers covering the research conducted on the use of natural compounds in dermatological diseases are also included in this Special Issue. Wah Wah Aung and his colleagues developed bioactive de-chlorophyll rhein-rich *Senna alata* extracts for improvements in cosmeceuticals and pharmaceuticals, and they demonstrated that a rhein-rich extract and its de-chlorophyll extracts possess antioxidant, anti-inflammatory, and antibacterial activities [3]. The results obtained by Schroder Verginica and her collaborators indicated a synergism between the mixture of clotrimazole and different citrus essential oils against *Staphylococcus aureus*, methicillin-resistant *Staphylococcus aureus* (MRSA), and *Pseudomonas aeruginosa*, microbial pathogens that are usually responsible for skin infections [4].

The study of antibacterial activities and other biological properties of essential oils was included in three other research articles. The essential oil extracted from the aerial parts of *Origanum elongatum* from Morocco exhibited remarkable antiradical and reducing power [5]. On the other hand, this essential oil was demonstrated to have antibacterial effects against *Enterococcus faecalis*, *Escherichia coli*, *Klebsiella oxytoca*, *Klebsiella pneumoniae*, *Enterobacter aerogenes*, *Serratia fonticola*, and *Acinetobacter baumannii*. In addition, the insecticidal activity on *Ceratitis capitata* adults recommends it as a biopesticide.

Aswathi Moothakoottil Kuttithodi and his colleagues evaluated the antioxidant properties of the essential oil obtained from *Cinnamommum malabatrum*, as well as other pharmacological effects, such as enzyme inhibition and antibacterial activity against different Gram-positive and Gram-negative bacteria [6].

The activity of 16 common essential oils on multidrug-resistant (MDR) *Pseudomonas aeruginosa* clinical isolates and the effect of the most potent one, cinnamon essential oil, on *mex* efflux pump gene expression was investigated by Razvan Lucian Coseriu and his team. They found that cinnamon essential oil had the best antibacterial activity, even at very low concentrations, while the activity of thyme, turmeric, peppermint, basil, clove, and lavender essential oils presented variable results. The results obtained using cinnamon essential oil by this team suggest the potential of this product to be used as an adjuvant in the antibacterial treatment of infections produced by multidrug-resistant strains of *Pseudomonas aeruginosa* [7]. 

The anticancer, antibacterial, anti-quorum-sensing, and antibiofilm effects of AgNPs synthesized from *Eruca sativa* leaf extracts are the center of attention in the work of Amir Mahgoub Awadelkareem and his coworkers [8]. In their study, they were able to demonstrate that a green nano-chemistry methodology can be effectively applied to produce metal nanostructures, which can be used in developing drugs and fabricating medical devices that resist colonization by antibiotic-resistant pathogenic bacteria.

Jinliang Du and coauthors investigated the protective effects of *Glycyrrhiza* total flavones (GTFs) on liver injury induced by *Streptococcus agalactiae* in fish [9]. Their conclusion was that the addition of GTFs to the diet could improve oxidative stress injury caused by *S. agalactiae* and repair the damaged tissue in *Oreochromis niloticus* liver.

This Special Issue brings together several papers showing the enormous therapeutic potential of plant extracts, both in terms of antioxidant effects, and especially antimicrobial effects on bacterial strains with increased resistance to antibiotics, which is considered to be a great challenge of this century.

## References

[1] Bora L., Avram S., Pavel I.Z., Muntean D., Liga S., Buda V., Gurgus D., Danciu C. (2022). An Up-To-Date Review Regarding Cutaneous Benefits of *Origanum vulgare* L. Essential Oil. Antibiotics.

[2] Häsler Gunnarsdottir S., Sommerauer L., Schnabel T., Oostingh G.J., Schuster A. (2023). Antioxidative and Antimicrobial Evaluation of Bark Extracts from Common European Trees in Light of Dermal Applications. Antibiotics.

[3] Aung W.W., Panich K., Watthanophas S., Naridsirikul S., Ponphaiboon J., Krongrawa W., Kulpicheswanich P., Limmatvapirat S., Limmatvapirat C. (2023). Preparation of Bioactive De-Chlorophyll Rhein-Rich *Senna alata* Extract. Antibiotics.

[4] Schroder V., Radu N., Cornea P.C., Coman O.A., Pirvu L.C., Mohammed M.S.O., Stefaniu A., Pintilie L., Bostan M., Caramihai M.D. (2022). Studies Regarding the Antimicrobial Behavior of Clotrimazole and Limonene. Antibiotics.

[5] Tagnaout I., Zerkani H., Bencheikh N., Amalich S., Bouhrim M., Mothana R.A., Alhuzani M.R., Bouharroud R., Hano C., Zair T. (2023). Chemical Composition, Antioxidants, Antibacterial, and Insecticidal Activities of *Origanum elongatum* (Bonnet) Emberger & Maire Aerial Part Essential Oil from Morocco. Antibiotics.

[6] Kuttithodi A.M., Narayanankutty A., Visakh N.U., Job J.T., Pathrose B., Olatunji O.J., Alfarhan A., Ramesh V. (2023). Chemical Composition of the *Cinnamomum malabatrum* Leaf Essential Oil and Analysis of Its Antioxidant, Enzyme Inhibitory and Antibacterial Activities. Antibiotics.

[7] Coșeriu R.L., Vintilă C., Pribac M., Mare A.D., Ciurea C.N., Togănel R.O., Cighir A., Simion A., Man A. (2023). Antibacterial Effect of 16 Essential Oils and Modulation of *mex* Efflux Pumps Gene Expression on Multidrug-Resistant *Pseudomonas aeruginosa* Clinical Isolates: Is Cinnamon a Good Fighter?. Antibiotics.

[8] Awadelkareem A.M., Al-Shammari E., Elkhalifa A.O., Adnan M., Siddiqui A.J., Patel M., Khan M.I., Mehmood K., Ashfaq F., Badraoui R. (2022). Biosynthesized Silver Nanoparticles from *Eruca sativa* Miller Leaf Extract Exhibits Antibacterial, Antioxidant, Anti-Quorum-Sensing, Antibiofilm, and Anti-Metastatic Activities. Antibiotics.

[9] Du J., Cao L., Gao J., Jia R., Zhu H., Nie Z., Xi B., Yin G., Ma Y., Xu G. (2022). Protective Effects of *Glycyrrhiza* Total Flavones on Liver Injury Induced by *Streptococcus agalactiae* in Tilapia (*Oreochromis niloticus*). Antibiotics.

